# *catena*-Poly[[diaqua­bis­[2-(4-tolyl­sulfanyl)acetato-κ*O*]manganese(II)]-μ-4,4′-bipyridine-κ^2^ *N*:*N*′]

**DOI:** 10.1107/S1600536811011263

**Published:** 2011-04-07

**Authors:** Xiao-Yong Zheng

**Affiliations:** a Wenzhou Medical College, Wenzhou, Zhejiang 325000, People’s Republic of China

## Abstract

In the polymeric title complex, [Mn(C_9_H_9_O_2_S)_2_(C_10_H_8_N_2_)(H_2_O)_2_]_*n*_, the Mn^2+^ cation and the 4,4′-bipyridine ligand lie on a twofold rotation axis. The cation has an MnN_2_O_4_ octa­hedral environment, being coordinated by the O atoms of two water mol­ecules and two monodentate (4-tolyl­sulfan­yl)acetate anions, and by two N atoms of two 4,4′-bipyridine ligands. The latter bridge adjacent cations into linear chains parallel to [010]. The chains are further linked with each other into a two-dimensional network parallel to (100) *via* inter­molecular O—H⋯O hydrogen bonds.

## Related literature

For isotypic structures, see: Cai *et al.* (2008 ▶) for the Cd, Lin *et al.* (2006 ▶) for the Ni, and Zheng *et al.* (2006 ▶) for the Co analogue.
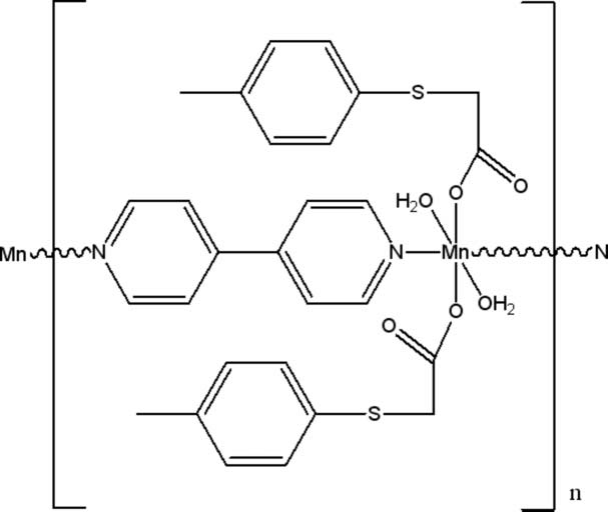


## Experimental

### 

#### Crystal data

[Mn(C_9_H_9_O_2_S)_2_(C_10_H_8_N_2_)(H_2_O)_2_]
*M* *_r_* = 609.60
Monoclinic, 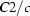

*a* = 21.750 (4) Å
*b* = 11.618 (2) Å
*c* = 11.028 (2) Å
β = 93.24 (3)°
*V* = 2782.4 (10) Å^3^
*Z* = 4
Mo *K*α radiation
μ = 0.67 mm^−1^
*T* = 293 K
0.33 × 0.28 × 0.27 mm

#### Data collection

Bruker APEXII CCD diffractometer
Absorption correction: multi-scan (*SADABS*; Sheldrick, 1996 ▶) *T*_min_ = 0.800, *T*_max_ = 0.835
12205 measured reflections
3129 independent reflections
2806 reflections with *I* > 2σ(*I*)
*R*_int_ = 0.016

#### Refinement

*R*[*F* ^2^ > 2σ(*F* ^2^)] = 0.034
*wR*(*F* ^2^) = 0.095
*S* = 1.07
3129 reflections
187 parameters
3 restraints
H atoms treated by a mixture of independent and constrained refinement
Δρ_max_ = 0.67 e Å^−3^
Δρ_min_ = −0.27 e Å^−3^

### 

Data collection: *APEX2* (Bruker, 2006 ▶); cell refinement: *SAINT* (Bruker, 2006 ▶); data reduction: *SAINT*; program(s) used to solve structure: *SHELXS97* (Sheldrick, 2008 ▶); program(s) used to refine structure: *SHELXL97* (Sheldrick, 2008 ▶); molecular graphics: *DIAMOND* (Brandenburg, 2006 ▶); software used to prepare material for publication: *SHELXL97*.

## Supplementary Material

Crystal structure: contains datablocks I, global. DOI: 10.1107/S1600536811011263/wm2465sup1.cif

Structure factors: contains datablocks I. DOI: 10.1107/S1600536811011263/wm2465Isup2.hkl

Additional supplementary materials:  crystallographic information; 3D view; checkCIF report

## Figures and Tables

**Table 1:** Selected bond lengths (Å)

Mn1—O1*W*	2.1843 (13)
Mn1—O2	2.1985 (12)
Mn1—N1	2.2593 (17)

**Table 2:** Hydrogen-bond geometry (Å, °)

*D*—H⋯*A*	*D*—H	H⋯*A*	*D*⋯*A*	*D*—H⋯*A*
O1*W*—H1*WB*⋯O2^i^	0.83 (2)	2.00 (2)	2.7934 (16)	161 (2)
O1*W*—H1*WA*⋯O1	0.83 (2)	1.87 (2)	2.6571 (19)	157 (2)

Symmetry code: (i) 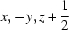.

**Fig. 1.**
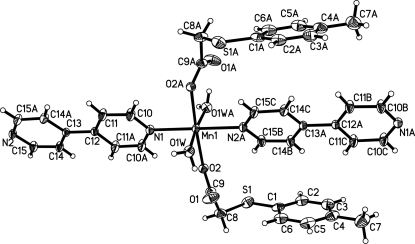
A view of part of the title structure, showing 30% probability displacement ellipsoids. [Symmetry codes: (A -x, y, -z + 1/2; B x, y + 1, z; C x, y - 1,z.]

**Fig. 2.**
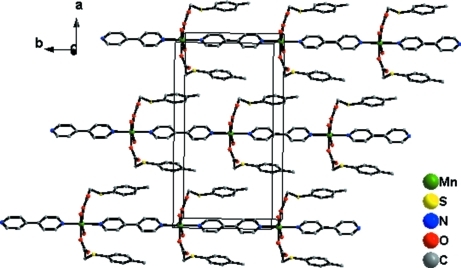
The chain structure of the title compound. All H atoms have been omitted for clarity.

## supplementary crystallographic information

### Comment

The title complex, (I), is isotypic with the Ni^2+^ (Lin *et al.*, 2006), Co^2+^ (Zheng *et al.*, 2006) and Cd^2+^ analogues (Cai *et al.*, 2008).

The structure of (I) (Fig. 1) consists of linear polymeric chains parallel to [010]. The chains are formed through 4,4'-bipyridine ligands bridging the Mn^2+^ cations. The latter are in a slightly distorted MnN_2_O_4_ octahedral coordination environment defined by four O atoms of pairs of water molecules and monodentate (4-tolylsulfanyl)acetate anions. Intermolecular O—H···O hydrogen bonding between the water molecules and the carboxylate O atoms link neighboring chains into a two-dimensional network parallel to (100).

In comparison with the isotypic structures, the structural parameters are very similar; as expected, the metal—N and metal—O bond lengths differ slightly due to the differences of the ionic radii of Cd^2+^, Co^2+^, Mn^2+^ and Ni^2+^.

### Experimental

Mn(CH_3_COO)_2_^.^4H_2_O (0.132 g, 0.5 mmol), (4-tolylsulfanyl)acetic acid (0.091 g, 0.5 mmol), 4,4'-bipyridine (0.039 g, 0.25 mmol) and water (18 ml) were sealed in a 25 ml Teflon-lined stainless-steel reactor. The solution was heated at 433 K for 72 h and then cooled to room temperature over a period of 72 h. Yellow crystals suitable for X-ray analysis were obtained.

### Refinement

The methyl groups were allowed to rotate with C—H = 0.96 Å and *U*_iso_(H) = 1.2*U*_eq_(C); the other H atoms were positioned geometrically [aromatic C—H = 0.93 Å and aliphatic C—H = 0.97 Å, *U*_iso_(H) = 1.2*U*_eq_(C)]. Water H atoms were located from difference Fourier maps and were refined with distance restraints of O—H = 0.85 Å and H···H = 1.30 Å; their displacement parameters were set to 1.2*U*_eq_(O).

### Crystal data

**Table:**

[Mn(C_9_H_9_O_2_S)_2_(C_10_H_8_N_2_)(H_2_O)_2_]	*F*(000) = 1268
*M**_r_* = 609.60	*D*_x_ = 1.455 Mg m^−^^3^
Monoclinic, *C*2/*c*	Mo *K*α radiation, λ = 0.71073 Å
Hall symbol: -C 2yc	Cell parameters from 6375 reflections
*a* = 21.750 (4) Å	θ = 2.7–27.4°
*b* = 11.618 (2) Å	µ = 0.67 mm^−^^1^
*c* = 11.028 (2) Å	*T* = 293 K
β = 93.24 (3)°	Prism, yellow
*V* = 2782.4 (10) Å^3^	0.33 × 0.28 × 0.27 mm
*Z* = 4	

### Data collection

**Table:**

Bruker APEXII CCD diffractometer	3129 independent reflections
Radiation source: fine-focus sealed tube	2806 reflections with *I* > 2σ(*I*)
graphite	*R*_int_ = 0.016
ω scans	θ_max_ = 27.4°, θ_min_ = 2.7°
Absorption correction: multi-scan (*SADABS*; Sheldrick, 1996)	*h* = −27→27
*T*_min_ = 0.800, *T*_max_ = 0.835	*k* = −14→14
12205 measured reflections	*l* = −14→14

### Refinement

**Table:**

Refinement on *F*^2^	Primary atom site location: structure-invariant direct methods
Least-squares matrix: full	Secondary atom site location: difference Fourier map
*R*[*F*^2^ > 2σ(*F*^2^)] = 0.034	Hydrogen site location: inferred from neighbouring sites
*wR*(*F*^2^) = 0.095	H atoms treated by a mixture of independent and constrained refinement
*S* = 1.07	*w* = 1/[σ^2^(*F*_o_^2^) + (0.0592*P*)^2^ + 1.086*P*] where *P* = (*F*_o_^2^ + 2*F*_c_^2^)/3
3129 reflections	(Δ/σ)_max_ = 0.001
187 parameters	Δρ_max_ = 0.67 e Å^−^^3^
3 restraints	Δρ_min_ = −0.27 e Å^−^^3^

### Special details

**Table:**

Geometry. All e.s.d.'s (except the e.s.d. in the dihedral angle between two l.s. planes) are estimated using the full covariance matrix. The cell e.s.d.'s are taken into account individually in the estimation of e.s.d.'s in distances, angles and torsion angles; correlations between e.s.d.'s in cell parameters are only used when they are defined by crystal symmetry. An approximate (isotropic) treatment of cell e.s.d.'s is used for estimating e.s.d.'s involving l.s. planes.
Refinement. Refinement of *F*^2^ against ALL reflections. The weighted *R*-factor *wR* and goodness of fit *S* are based on *F*^2^, conventional *R*-factors *R* are based on *F*, with *F* set to zero for negative *F*^2^. The threshold expression of *F*^2^ > σ(*F*^2^) is used only for calculating *R*-factors(gt) *etc*. and is not relevant to the choice of reflections for refinement. *R*-factors based on *F*^2^ are statistically about twice as large as those based on *F*, and *R*- factors based on ALL data will be even larger.

### Fractional atomic coordinates and isotropic or equivalent isotropic displacement parameters (Å^2^)

**Table:**

	*x*	*y*	*z*	*U*_iso_*/*U*_eq_	
Mn1	0.0000	0.04840 (2)	0.2500	0.02718 (11)	
S1	0.14740 (2)	0.20715 (5)	−0.05268 (4)	0.05323 (15)	
N1	0.0000	−0.14607 (14)	0.2500	0.0327 (4)	
N2	0.0000	−0.75618 (14)	0.2500	0.0308 (3)	
O1W	0.06150 (6)	0.05814 (10)	0.41324 (10)	0.0408 (3)	
O1	0.15201 (6)	0.11457 (15)	0.27247 (12)	0.0670 (4)	
O2	0.07976 (5)	0.05427 (8)	0.13627 (10)	0.0351 (2)	
C1	0.16178 (7)	0.34725 (17)	0.00465 (15)	0.0451 (4)	
C2	0.14383 (8)	0.43759 (18)	−0.07231 (16)	0.0504 (4)	
H2A	0.1245	0.4218	−0.1478	0.060*	
C3	0.15447 (9)	0.55104 (18)	−0.03766 (18)	0.0556 (5)	
H3A	0.1416	0.6102	−0.0900	0.067*	
C4	0.18407 (9)	0.57787 (19)	0.07401 (17)	0.0553 (5)	
C5	0.20159 (9)	0.4866 (2)	0.14879 (17)	0.0584 (5)	
H5A	0.2217	0.5025	0.2236	0.070*	
C6	0.19070 (8)	0.3737 (2)	0.11752 (16)	0.0535 (5)	
H6A	0.2025	0.3151	0.1713	0.064*	
C7	0.19602 (12)	0.7009 (2)	0.1120 (2)	0.0775 (7)	
H7A	0.1808	0.7517	0.0485	0.116*	
H7B	0.2395	0.7125	0.1269	0.116*	
H7C	0.1753	0.7168	0.1848	0.116*	
C8	0.17674 (8)	0.11137 (18)	0.06561 (16)	0.0509 (4)	
H8A	0.1857	0.0375	0.0295	0.061*	
H8B	0.2152	0.1423	0.1004	0.061*	
C9	0.13315 (7)	0.09210 (14)	0.16781 (14)	0.0395 (3)	
C10	−0.02740 (9)	−0.20527 (13)	0.33381 (16)	0.0482 (4)	
H10A	−0.0469	−0.1649	0.3935	0.058*	
C11	−0.02857 (9)	−0.32390 (13)	0.33721 (15)	0.0446 (4)	
H11A	−0.0486	−0.3616	0.3981	0.054*	
C12	0.0000	−0.38670 (15)	0.2500	0.0266 (4)	
C13	0.0000	−0.51414 (16)	0.2500	0.0276 (4)	
C14	0.02495 (7)	−0.57707 (13)	0.15769 (14)	0.0377 (3)	
H14A	0.0426	−0.5393	0.0939	0.045*	
C15	0.02350 (8)	−0.69595 (13)	0.16070 (14)	0.0389 (3)	
H15A	0.0398	−0.7361	0.0970	0.047*	
H1WA	0.0956 (8)	0.0744 (18)	0.3876 (19)	0.064 (7)*	
H1WB	0.0694 (9)	0.0118 (17)	0.4691 (17)	0.060 (6)*	

### Atomic displacement parameters (Å^2^)

**Table:**

	*U*^11^	*U*^22^	*U*^33^	*U*^12^	*U*^13^	*U*^23^
Mn1	0.03535 (18)	0.02069 (17)	0.02614 (17)	0.000	0.00737 (12)	0.000
S1	0.0562 (3)	0.0687 (3)	0.0356 (2)	−0.0121 (2)	0.00958 (18)	0.00533 (19)
N1	0.0397 (9)	0.0278 (8)	0.0313 (8)	0.000	0.0069 (7)	0.000
N2	0.0386 (9)	0.0230 (8)	0.0316 (8)	0.000	0.0081 (7)	0.000
O1W	0.0475 (7)	0.0457 (7)	0.0293 (5)	−0.0007 (5)	0.0034 (5)	0.0052 (4)
O1	0.0484 (7)	0.1124 (12)	0.0401 (7)	−0.0236 (8)	0.0019 (5)	0.0076 (7)
O2	0.0367 (5)	0.0357 (6)	0.0338 (5)	−0.0019 (4)	0.0108 (4)	−0.0030 (4)
C1	0.0324 (8)	0.0698 (11)	0.0339 (8)	−0.0073 (7)	0.0097 (6)	0.0037 (7)
C2	0.0431 (9)	0.0744 (13)	0.0337 (8)	−0.0040 (8)	0.0032 (7)	0.0023 (8)
C3	0.0524 (11)	0.0698 (13)	0.0455 (10)	0.0008 (9)	0.0106 (8)	0.0033 (8)
C4	0.0450 (9)	0.0745 (12)	0.0481 (10)	−0.0094 (9)	0.0168 (8)	−0.0092 (9)
C5	0.0466 (10)	0.0917 (15)	0.0373 (9)	−0.0111 (10)	0.0053 (7)	−0.0085 (10)
C6	0.0440 (9)	0.0824 (14)	0.0344 (8)	−0.0030 (9)	0.0048 (7)	0.0060 (8)
C7	0.0748 (15)	0.0851 (17)	0.0749 (15)	−0.0180 (13)	0.0249 (12)	−0.0183 (13)
C8	0.0378 (8)	0.0680 (12)	0.0483 (10)	0.0040 (8)	0.0147 (7)	0.0096 (8)
C9	0.0355 (8)	0.0449 (9)	0.0389 (8)	0.0017 (6)	0.0082 (6)	0.0060 (7)
C10	0.0746 (12)	0.0263 (8)	0.0468 (9)	0.0013 (7)	0.0300 (8)	−0.0036 (6)
C11	0.0692 (11)	0.0258 (7)	0.0417 (8)	−0.0008 (7)	0.0284 (8)	0.0014 (6)
C12	0.0304 (9)	0.0213 (8)	0.0281 (9)	0.000	0.0030 (7)	0.000
C13	0.0311 (9)	0.0225 (9)	0.0294 (9)	0.000	0.0038 (7)	0.000
C14	0.0544 (9)	0.0258 (7)	0.0350 (8)	0.0026 (6)	0.0203 (7)	0.0035 (6)
C15	0.0570 (9)	0.0259 (7)	0.0355 (7)	0.0048 (6)	0.0181 (7)	0.0002 (6)

### Geometric parameters (Å, °)

**Table:**

Mn1—O1W	2.1843 (13)	C4—C5	1.383 (3)
Mn1—O1W^i^	2.1843 (13)	C4—C7	1.508 (3)
Mn1—O2	2.1985 (12)	C5—C6	1.373 (3)
Mn1—O2^i^	2.1985 (12)	C5—H5A	0.9300
Mn1—N1	2.2593 (17)	C6—H6A	0.9300
Mn1—N2^ii^	2.2704 (17)	C7—H7A	0.9600
S1—C1	1.768 (2)	C7—H7B	0.9600
S1—C8	1.8037 (19)	C7—H7C	0.9600
N1—C10	1.3209 (18)	C8—C9	1.530 (2)
N1—C10^i^	1.3209 (18)	C8—H8A	0.9700
N2—C15^i^	1.3333 (17)	C8—H8B	0.9700
N2—C15	1.3333 (17)	C10—C11	1.379 (2)
N2—Mn1^iii^	2.2704 (17)	C10—H10A	0.9300
O1W—H1WA	0.830 (15)	C11—C12	1.3824 (18)
O1W—H1WB	0.829 (15)	C11—H11A	0.9300
O1—C9	1.231 (2)	C12—C11^i^	1.3824 (18)
O2—C9	1.2717 (19)	C12—C13	1.481 (3)
C1—C2	1.392 (3)	C13—C14	1.3885 (17)
C1—C6	1.397 (3)	C13—C14^i^	1.3885 (17)
C2—C3	1.388 (3)	C14—C15	1.382 (2)
C2—H2A	0.9300	C14—H14A	0.9300
C3—C4	1.392 (3)	C15—H15A	0.9300
C3—H3A	0.9300		
			
O1W—Mn1—O1W^i^	174.06 (6)	C6—C5—H5A	118.5
O1W—Mn1—O2	90.14 (5)	C4—C5—H5A	118.5
O1W^i^—Mn1—O2	89.68 (5)	C5—C6—C1	119.79 (19)
O1W—Mn1—O2^i^	89.68 (5)	C5—C6—H6A	120.1
O1W^i^—Mn1—O2^i^	90.14 (5)	C1—C6—H6A	120.1
O2—Mn1—O2^i^	176.44 (5)	C4—C7—H7A	109.5
O1W—Mn1—N1	92.97 (3)	C4—C7—H7B	109.5
O1W^i^—Mn1—N1	92.97 (3)	H7A—C7—H7B	109.5
O2—Mn1—N1	91.78 (3)	C4—C7—H7C	109.5
O2^i^—Mn1—N1	91.78 (3)	H7A—C7—H7C	109.5
O1W—Mn1—N2^ii^	87.03 (3)	H7B—C7—H7C	109.5
O1W^i^—Mn1—N2^ii^	87.03 (3)	C9—C8—S1	114.49 (12)
O2—Mn1—N2^ii^	88.22 (3)	C9—C8—H8A	108.6
O2^i^—Mn1—N2^ii^	88.22 (3)	S1—C8—H8A	108.6
N1—Mn1—N2^ii^	180.0	C9—C8—H8B	108.6
C1—S1—C8	105.14 (9)	S1—C8—H8B	108.6
C10—N1—C10^i^	117.24 (18)	H8A—C8—H8B	107.6
C10—N1—Mn1	121.38 (9)	O1—C9—O2	125.49 (15)
C10^i^—N1—Mn1	121.38 (9)	O1—C9—C8	118.19 (15)
C15^i^—N2—C15	116.68 (17)	O2—C9—C8	116.32 (14)
C15^i^—N2—Mn1^iii^	121.66 (9)	N1—C10—C11	123.32 (14)
C15—N2—Mn1^iii^	121.66 (9)	N1—C10—H10A	118.3
Mn1—O1W—H1WA	104.3 (16)	C11—C10—H10A	118.3
Mn1—O1W—H1WB	132.1 (15)	C10—C11—C12	119.92 (14)
H1WA—O1W—H1WB	104.5 (17)	C10—C11—H11A	120.0
C9—O2—Mn1	126.33 (10)	C12—C11—H11A	120.0
C2—C1—C6	118.29 (18)	C11—C12—C11^i^	116.29 (18)
C2—C1—S1	116.00 (13)	C11—C12—C13	121.85 (9)
C6—C1—S1	125.68 (15)	C11^i^—C12—C13	121.85 (9)
C3—C2—C1	120.76 (17)	C14—C13—C14^i^	116.45 (17)
C3—C2—H2A	119.6	C14—C13—C12	121.78 (9)
C1—C2—H2A	119.6	C14^i^—C13—C12	121.78 (9)
C2—C3—C4	121.2 (2)	C15—C14—C13	119.89 (13)
C2—C3—H3A	119.4	C15—C14—H14A	120.1
C4—C3—H3A	119.4	C13—C14—H14A	120.1
C5—C4—C3	117.0 (2)	N2—C15—C14	123.54 (14)
C5—C4—C7	121.6 (2)	N2—C15—H15A	118.2
C3—C4—C7	121.5 (2)	C14—C15—H15A	118.2
C6—C5—C4	122.98 (18)		
			
O1W—Mn1—N1—C10	69.60 (11)	C2—C1—C6—C5	−1.1 (3)
O1W^i^—Mn1—N1—C10	−110.40 (11)	S1—C1—C6—C5	177.01 (13)
O2—Mn1—N1—C10	159.83 (11)	C1—S1—C8—C9	81.32 (15)
O2^i^—Mn1—N1—C10	−20.17 (11)	Mn1—O2—C9—O1	12.4 (2)
O1W—Mn1—N1—C10^i^	−110.40 (11)	Mn1—O2—C9—C8	−167.18 (11)
O1W^i^—Mn1—N1—C10^i^	69.60 (11)	S1—C8—C9—O1	−123.02 (17)
O2—Mn1—N1—C10^i^	−20.17 (11)	S1—C8—C9—O2	56.6 (2)
O2^i^—Mn1—N1—C10^i^	159.83 (11)	C10^i^—N1—C10—C11	−0.05 (15)
O1W—Mn1—O2—C9	−21.00 (12)	Mn1—N1—C10—C11	179.95 (15)
O1W^i^—Mn1—O2—C9	153.06 (12)	N1—C10—C11—C12	0.1 (3)
N1—Mn1—O2—C9	−113.98 (12)	C10—C11—C12—C11^i^	−0.05 (14)
N2^ii^—Mn1—O2—C9	66.02 (12)	C10—C11—C12—C13	179.95 (14)
C8—S1—C1—C2	178.98 (13)	C11—C12—C13—C14	175.42 (12)
C8—S1—C1—C6	0.82 (17)	C11^i^—C12—C13—C14	−4.58 (12)
C6—C1—C2—C3	0.1 (3)	C11—C12—C13—C14^i^	−4.58 (12)
S1—C1—C2—C3	−178.26 (14)	C11^i^—C12—C13—C14^i^	175.42 (12)
C1—C2—C3—C4	0.9 (3)	C14^i^—C13—C14—C15	0.57 (12)
C2—C3—C4—C5	−0.7 (3)	C12—C13—C14—C15	−179.43 (12)
C2—C3—C4—C7	179.74 (18)	C15^i^—N2—C15—C14	0.62 (13)
C3—C4—C5—C6	−0.4 (3)	Mn1^iii^—N2—C15—C14	−179.38 (13)
C7—C4—C5—C6	179.16 (18)	C13—C14—C15—N2	−1.2 (2)
C4—C5—C6—C1	1.3 (3)		

Symmetry codes: (i) −*x*, *y*, −*z*+1/2; (ii) *x*, *y*+1, *z*; (iii) *x*, *y*−1, *z*.

### Hydrogen-bond geometry (Å, °)

**Table:**

*D*—H···*A*	*D*—H	H···*A*	*D*···*A*	*D*—H···*A*
O1W—H1WB···O2^iv^	0.83 (2)	2.00 (2)	2.7934 (16)	161 (2)
O1W—H1WA···O1	0.83 (2)	1.87 (2)	2.6571 (19)	157 (2)

Symmetry codes: (iv) *x*, −*y*, *z*+1/2.
